# Intratumoral pseudoaneurysm within a liver metastasis of gastric cancer: a case report

**DOI:** 10.1186/s40792-020-00806-z

**Published:** 2020-02-18

**Authors:** Hiroki Ohara, Yuji Ishibashi, Shuntaro Yoshimura, Ryoto Yamazaki, Fumihiko Hatao, Takeshi Koshiishi, Yasuhiro Morita, Kazuhiro Imamura

**Affiliations:** 1 Department of Surgery, Tokyo Metropolitan Tama Medical Center, 2-8-29 Musashidai, Fuchu-shi, Tokyo, 183-8524 Japan; 2 Department of Surgery, Tokyo Metropolitan Matsuzawa Hospital, 2-1-1 Kamikitazawa, Setagaya-ku, Tokyo, 156-0057 Japan; 3 Department of Radiology, Tokyo Metropolitan Tama Medical Center, 2-8-29 Musashidai, Fuchu-shi, Tokyo, 183-8524 Japan

**Keywords:** Pseudoaneurysm, Hepatic artery, Gastric cancer, Metastatic liver tumor

## Abstract

**Background:**

Intrahepatic artery pseudoaneurysms are mostly iatrogenic and result from hepatobiliary interventions. The incidence of intrahepatic artery pseudoaneurysms within liver tumors without prior intervention is extremely rare. We presented herein the first report of a case of an intratumoral pseudoaneurysm within a liver metastasis of gastric cancer without any prior intervention during chemotherapy.

**Case presentation:**

A 59-year-old male patient underwent a distal gastrectomy and D2 lymph node dissection for gastric cancer. He was treated in the emergency room for right abdominal pain following the 4th cycle of nivolumab administration as second-line chemotherapy after adjuvant chemotherapy with S-1 and first-line chemotherapy for a liver metastasis of gastric cancer with ramucirumab plus paclitaxel. CT showed a 72-mm metastatic liver tumor containing a 9-mm pseudoaneurysm and fluid collection around the hepatic edge. Intrahepatic artery pseudoaneurysm within the metastatic liver tumor was diagnosed, with the surrounding fluid indicating potential, active bleeding. An emergency angiography confirmed the presence of a pseudoaneurysm in the intrahepatic artery, which was embolized using microcoils. The contributory causes of the intratumoral pseudoaneurysm were assumed to be the following: (1) tumor necrosis leading to encasement, erosion of the vessel wall, and subsequent arterial wall weakening; and (2) inhibition of vascular endothelial growth by ramucirumab resulting in a vessel wall breach and pseudoaneurysm formation.

**Conclusion:**

It is necessary to recognize that pseudoaneurysms can arise within a metastatic liver tumor during chemotherapy.

## Introduction

Pseudoaneurysms result from the partial to complete disruption of the vascular wall and lead to hemorrhaging contained by the adventitia of the vessel wall or the perivascular soft tissues [1]. Intrahepatic artery pseudoaneurysms are rare and mostly iatrogenic, resulting from hepatobiliary interventions. The incidence of pseudoaneurysms within a liver tumor without prior intervention is extremely rare. We presented herein the first report of a case of intratumoral pseudoaneurysm (ITPA) within a liver metastasis of gastric cancer during chemotherapy without any prior intervention.

## Case report

**Fig. 1:**
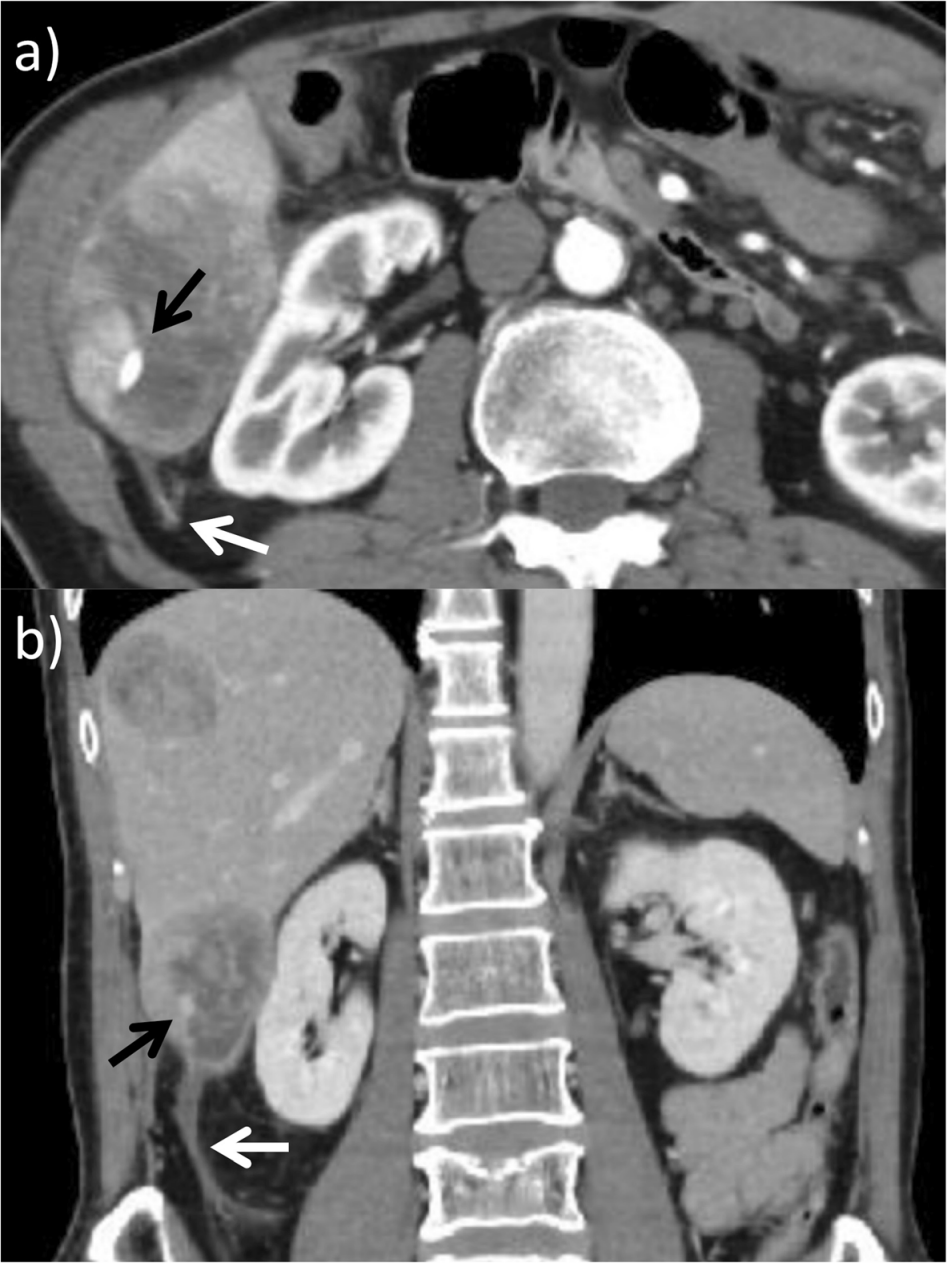
**a**, **b** CT revealed a 72 × 68 mm metastatic liver tumor in segment VI containing a 9-mm pseudoaneurysm (black arrow) and fluid collection around the hepatic edge (white arrow)

**Fig. 2:**
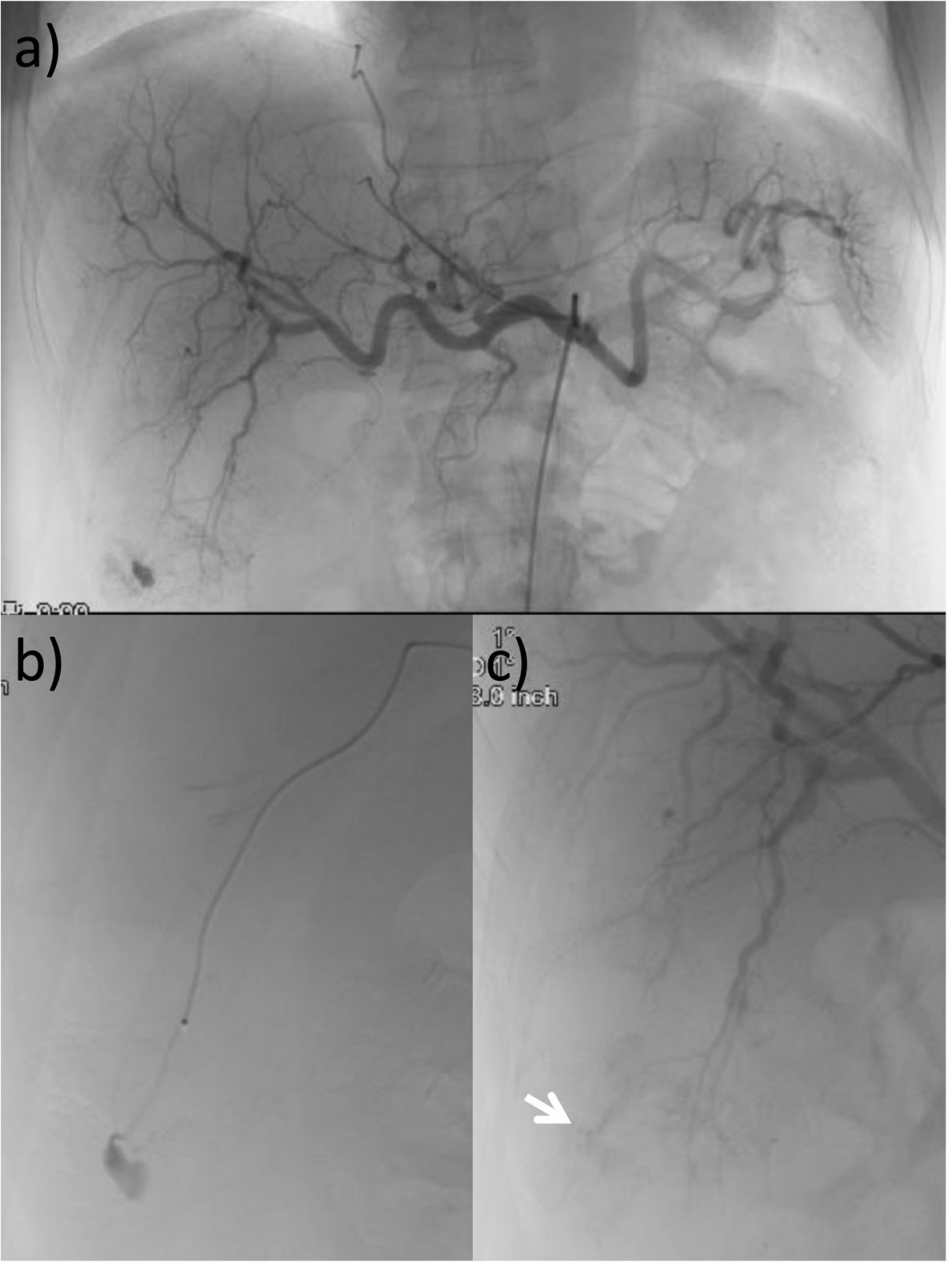
**a**, **b** Emergency angiography showed a pseudoaneurysm stemming from a branch of the hepatic artery in segment VI. **c** The pseudoaneurysm was cannulated and successfully embolized using microcoils (white arrow)

A 59-year-old male patient underwent a distal gastrectomy and D2 lymph node dissection for gastric cancer. The final pathological stage was T4aN3aM0 Stage IIIC according to the Japanese Gastric Cancer Association Classification, 14th Edition [2]. Postoperative adjuvant chemotherapy with S-1 (100 mg/day p.o., twice daily for 28 consecutive days) was administered for 1 year in accordance with treatment guidelines. One and a half years after surgery, computed tomography (CT) revealed a liver metastasis in hepatic segment VI. The recurrence was detected within 6 months after adjuvant chemotherapy with S-1, and combination therapy with ramucirumab (RAM: 8 mg/kg on days 1 and 15) and paclitaxel (PTX: 80 mg/m2 on days 1, 8, and 15) was administered as first-line chemotherapy, with the regimen repeated at 28-day intervals. After eight cycles with RAM plus PTX, follow-up CT revealed a new liver metastasis in segments VI and VIII. Nivolumab (3 mg/kg, biweekly) was administered as second-line treatment. Five days after the 4th cycle of nivolumab injections, the patient visited our emergency room due to right abdominal pain. Guarding and rebound tenderness were denied. On initial physical examination, the blood pressure was 100/64 mmHg and the heart rate was 109 beats per min. Serum biochemistry showed a white blood cell count of 8.1 × 10^3^/μL, red blood cell count of 478 × 10^4^/μL, and hemoglobin 14.2 g/dL. CT showed a 72 × 68 mm metastatic liver tumor in segment VI. The tumor rim was enhanced, but the center was not, and thus considered to consist of necrotic tissue. The tumor contained a 9-mm pseudoaneurysm, and fluid collection was visible around hepatic edge (Fig. 1). An intrahepatic artery pseudoaneurysm within the metastatic liver tumor was diagnosed, with the surrounding fluid indicating potential, active bleeding. An emergency angiography confirmed the presence of a pseudoaneurysm stemming from a branch of the hepatic artery in segment VI (Fig. 2). The pseudoaneurysm was cannulated and successfully embolized using microcoils. After embolization, there were no clinical signs, and the patient was discharged 9 days after the angiography without any recurrence of bleeding. Follow-up CT 1 month after the angiography detected enlarged liver and bone metastasis. In accordance with the wishes of the patient and his family, palliative care was begun. The patient remains alive with palliative care 5 months after the angiography.

## Discussion

In this rare case, ITPA within a liver metastasis of gastric cancer following chemotherapy without any prior hepatobiliary intervention was ruptured and treated using arterial embolization.

Visceral artery aneurysms are rare. Aneurysms of the hepatic artery, celiac axis, and branches, and splenic artery have a prevalence of 39%, 39%, and 18%, respectively [3]. Of cases of hepatic artery aneurysm, 77% are confined to the segment proximal to the liver, 20% have combined intra- and extraparenchymal involvement, and 3% are localized exclusively within the liver [4]. The present case was a pseudoaneurysm arising exclusively from the intrahepatic artery (branch of the segment VI hepatic artery).

The causes of visceral artery pseudoaneurysms are trauma, infection, inflammatory diseases, and complications of abdominal surgery, hepatobiliary interventions, and endoscopic explorations [5]. On the other hand, the incidence of pseudoaneurysms caused by malignant tumors is low although pseudoaneurysms caused by hepatocellular carcinomas (HCC), malignant lymphomas, desmoids, neurofibromatosis, giant cell tumors, leukemia, and so on have been reported [6–13].

An ITPA arising from the intrahepatic artery within a liver tumor is rare. While some cases of ITPA in the liver have been reported, almost all these tumors were hepatocellular carcinomas (HCC) [6–8, 14, 15]. ITPA within a liver metastasis of gastric cancer is extremely rare (Table 1). Liu et al. reported such a case caused by vascular injury after radiofrequency ablation (RFA) [16].

**Table 1:** Clinical characteristics of patients with ITPA within a liver metastasis of gastric cancer

Author	Gender	Age	Symptom	Prior intervention	Tumor size (mm)	Site	ITPA size (mm)	Treatment	Outcome
Liu [16]	M	77	Hematemesis	RFA	45	Segment III	Not described	Embolization	Alive
Our case	M	59	Abdominal pain	none	72	Segment VI	9	Embolization	Alive

*ITPA* intratumoral pseudoaneurysm, *RFA* radiofrequency ablation

Intrahepatic artery pseudoaneurysms are iatrogenic, resulting from hepatobiliary intervention [17]. Even in ITPA, hepatobiliary interventions like transcatheter arterial chemoembolization and RFA resulting in vascular catastrophe are the most common causes [14–16, 18]. Yoshikawa et al. reported a case of ITPA within an HCC after carbon ion radiotherapy and described the cause as being angiogenesis, fragmentation of the vascular mesothelial elastic fibers, and edema of the subcutaneous blood vessels due to radiation [8]. However, few researchers have reported pseudoaneurysms arising de novo from within a HCC without any prior intervention [6, 7]. Among these, Haider et al. reported a case series (including 25 cases) and an annual incidence of 0.24% for the condition. The development of pseudoaneurysms is thought to be related to tumor angiogenesis [7].

In the present case, the patient had no hypertension, cardiovascular history, inflammatory disease, or recent trauma, and the metastatic liver tumor had not been treated by any hepatobiliary or other surgical procedure or radiation. Anatomical abnormalities, such as vascular malformation and tumor angiogenesis, were not observed on either the CT or angiogram. The cause of the ITPA in the present case was unclear, but the tumor necrosis found on CT likely led to encasement, erosion of the vessel wall, and subsequent arterial wall weakening leading to the development of the pseudoaneurysm as the tumor progressed. The chemotherapeutic drugs administered to the patient may have contributed to the conditions favoring its development. Two cases of pseudoaneurysm developing after FOLFIRI (irinotecan, leucovorin, 5-fluorouracil: 5-FU) combined with bevacizmab and FOLFOX (oxaliplatin, leucovorin, 5-FU) have been reported [19, 20]. Two other cases involved acute enlargement of an abdominal aortic aneurysm following chemotherapy with gemcitabine, cisplatin, docetaxel, and 5-FU [21, 22]. Some chemotherapy drugs have vascular toxicity and induce cell apoptosis leading to loss of integrity of the vascular wall [19–23]. In the present case, the ITPA was diagnosed during nivolumab administration as second-line chemotherapy following S-1 as adjuvant chemotherapy and RAM plus PTX as first-line chemotherapy. A previous study reported that 5-FU, its oral pro-drug, and PTX primarily alter the molecular signaling pathways controlling vascular smooth muscle cell tone, thereby inducing vasoconstriction, but did not explain the relationship of these drugs to pseudoaneurysm formation [23]. RAM is a monoclonal antibody that binds to vascular endothelial growth factor (VEGF) receptor-2, preventing its activation. Bleeding is a major adverse event reported in some clinical trials caused by angiogenesis inhibitors disrupting the tumor vasculature by inhibiting VEGF signaling, leading to thrombosis or bleeding [24, 25]. RAM may inhibit endothelial growth, thus resulting in a vessel wall breach and pseudoaneurysm formation. However, due to the absence of reports of similar cases, it is unclear whether any correlation exists between RAM and ITPA formation. However, such a correlation cannot be entirely ruled out, and it is possible that RAM is responsible for the bleeding caused by an ITPA rupture.

The prevalence of hepatic artery pseudoaneurysm ruptures can be as high as 90% [26]. As patients with visceral artery aneurysm rupture frequently present with hemorrhagic shock, prompt resuscitation with blood products and hemorrhage control are critical [27]. There are several effective approaches for treating hepatic artery pseudoaneurysms, including open surgery, which has a 21% mortality rate, as well as endovascular methods, which have a low complication and mortality rate [28]. Recent interventions using arterial embolization or stent grafts have been proposed as alternatives to surgical repair and offer real advantages in terms of survival [3].

## Conclusion

We reported a case of intratumoral pseudoaneurysm within a liver metastasis of gastric cancer detected during chemotherapy. It is necessary to recognize that pseudoaneurysms can arise within a metastatic liver tumor during chemotherapy.

## Data Availability

The data are not available for public access due to patient privacy concerns but are available from the corresponding author on reasonable request.

## Abbreviations

CT: Computed tomography
FOLFIRI: Irinotecan, leucovorin, 5-fluorouracil
FOLFOX: Oxaliplatin, leucovorin, 5-FU
HCC: Hepatocellular carcinoma
ITPA: Intratumoral pseudoaneurysm
PTX: Paclitaxel
RAM: Ramucirumab
RFA: Radiofrequency ablation
VEGF: Vascular endothelial growth factor
